# Diversity Shifts in the Root Microbiome of Cucumber Under Different Plant Cultivation Substrates

**DOI:** 10.3389/fmicb.2022.878409

**Published:** 2022-05-19

**Authors:** Fangyuan Zhou, Xiaoqing Wu, Yunxiao Gao, Susu Fan, Hongzi Zhou, Xinjian Zhang

**Affiliations:** Shandong Provincial Key Laboratory of Applied Microbiology, Ecology Institute, Qilu University of Technology (Shandong Academy of Sciences), Ji’nan, China

**Keywords:** root microbiota, high throughput sequencing, soilless cultivation, microflora, plant-microbe interaction

## Abstract

Application of plant artificial cultivation substrates lead to alteration of rhizosphere environment. Whether this alteration could lead to root microbiome variation was limitedly investigated. This work aims to determine the diversity shifts in the root microbiome of cucumber under different plant cultivation substrates and predict corresponding function of these different root bacterial microbiota. Cucumber root samples cultivated with two artificial cultivation substrates and greenhouse soils were prepared. Subsequently, high throughput sequencing and bioinformatics analysis were applicated to compare the root bacterial diversity of cucumber cultivated in different substrates and their corresponding function. In total, 311,039 sequences were obtained, and they were annotated to 42 operational taxonomic units (OTUs), belonging to 28 genera, 18 families, 12 orders, four classes, and three phyla. The *α* and *β* diversity of samples from the two cultivation substrates and greenhouse soils were significantly different. Only 2–3 bacterial species were found to be discrepancy between cucumber root samples from artificial cultivation substrates and from greenhouse soils. The relative abundance of genus *Asticcacaulis*, *Methylophilus*, *Massilia*, *Dyella*, and *Devosia* in samples of artificial cultivation substrates was significantly higher than that of soils, while the relative abundance of genus *Phenylobacterium*, *Noviherbaspirillum*, and *Arenimonas* was significantly lower than that of soils. Besides, compared to cucumber root bacterial community cultivated in soils, the abundance of synthetic pathways for flavonoids and flavonols, bile acids, indole alkaloids, lactose, and neolactose increased by 41.6-, 28.7-, 5.9-, and 5.5-fold, respectively, in the bacterial community of the substrate 1-cultivated roots, and the abundance of clavulanic acid, receptor interaction, sesquiterpenoid, bile acid, flavonoid and flavonol, indole alkaloid, lactose, and neolactose synthetic pathways increased by 42.3-, 32.4-, 32.4-, 13.9-, 10.3-, 6.3-, and 5.2-fold, respectively, in the bacterial community of the substrate two-cultivated roots. This paper verified the diversity shifts in the root microbiome of cucumber under different plant cultivation substrates. Besides, the corresponding function difference of these different root bacterial microbiota was predicted. This work would provide theoretical support for discovering microbial resources and building artificial microbial flora.

## Introduction

During long time of coevolution, plants have formed a close symbiotic relationship with microorganisms. These microorganisms inhabit on surface of plant roots and leaves as well as within plant tissues performing important ecological functions (Berendsen et al., 2012). For example, certain rhizosphere microorganisms play important roles in nutrient uptake and in assisting plants to resist adverse environments (Shaikh et al., 2018). Besides, root microbes contribute to phytopathogen resistance of host plants (Kwak et al., 2018). Among those microorganisms, bacteria constitute a large percentage of the various plant rhizosphere and endophyte microbial communities (Hassan et al., 2019) and are currently receiving extensive attention from researchers worldwide. Plant root bacteria have been reported to improve crop productivity and resistance in an increasing number of studies (de Vries et al., 2020; Ling et al., 2022; Pronk et al., 2022), which in turn has prompted researches on root microbial diversity in crops.

Previous studies on the diversity of crop root microorganisms have revealed the effects of factors such as fertilizers, soil nutrients, soil types, and root diseases on crop microbial diversity (Yang et al., 2011; Philippot et al., 2013; Kwak et al., 2018; Qin et al., 2019). These studies have mainly focused on the changes in the root microbial diversity of crop cultivated in field soils (Fang et al., 2019). However, with the development of modern agriculture, crop cultivation techniques are constantly updated, and crop cultivation substrates that have a direct impact on the composition of root microorganisms are no longer limited to natural field soils. New synthetic substrates for crop cultivation are continually developed and applicated, and those artificial substrates support efficient and intensive plant production since then (Choi et al., 2012; Barrett et al., 2016; Awad et al., 2017). In contrast, studies on the root microbial diversity of crops cultivated in artificial substrates were rarely conducted (Nie et al., 2014). Whether the composition of microorganisms enriched in the rhizosphere of crops will alter in the new cultivation substrate or what functional changes the microbial composition will bring was unknown. Besides, researchers have begun to construct the root microbiome of plants to reproduce the microflora function at the community level (Liu et al., 2019; Zhang et al., 2021), which makes it more necessary to investigate the shifts in root microbiome of crops under different plant cultivation substrates.

Cucumber, *Cucumis sativus* L., is an important economic crop in China. It is planted in a large scale and brings high economic benefits each year (Ma et al., 2021). Currently, its cultivation is primarily dependent on greenhouses owing to higher retail price compared to seasonal outdoor vegetables (Xu, 2019). With the advancement of cultivation technology, more and more farmers are opting to grow cucumbers using artificial cultivation substrates (Gül et al., 2007; Vukobratović et al., 2016; Sarwar et al., 2018), which not only reduces the occurrence of cucumber pests and root diseases but also promotes the growth of cucumber roots and fertilizer utilization (Kraska et al., 2018; Sarwar et al., 2018; Shi et al., 2020). As it has been reported in other crops (Hernández and Hobbie, 2010; Frouz et al., 2016) and cucumber (Tian and Gao, 2014), the alteration in cultivation substrates mentioned above would inevitably bring tremendous effects on root microbial community of cucumber. However, the root microbial community difference of cucumber cultivated in field soils and artificial cultivation substrates was scarcely investigated. Thus, we hypothesized that the root microbial community of cucumber would shift under different plant cultivation substrates, and the corresponding function would also change. Basing on this hypothesis, two types of artificial cultivation substrates and one greenhouse soil, which are commonly used in cucumber cultivation and have different major components, were selected as cultivation substrates to compare the root bacterial composition of cucumber under different cultivation substrates in this study. Besides, the function of cucumber root microbiota under various growing substrates was predicted and compared. This study would provide a theoretical basis for exploring beneficial microorganisms from the cucumber root microbiome and constructing artificial root microflora.

## Materials and Methods

### Materials

Cucumber variety: Cucumber variety Qiangsheng 719 produced by the Shandong Lushou Agricultural Company. Three types of cultivation substrates including greenhouse soils and two artificial plant cultivation substrates were used in this study. Specifically, the greenhouse soil (GHS, Organic Matter: 29.8 g kg^−1^, Total N: 1.68 g kg^−1^, NH_4_^+^-N: 6.64 mg kg^−1^, NO_3_^−^-N: 48.75 mg kg^−1^, Olsen-P: 132.67 mg kg^−1^, NH_4_OAc-K: 456.63 mg kg^−1^, pH: 7.02, and EC: 0.48 mS cm^−1^) was collected from the No. 1 greenhouse in the experimental base of Shandong Shangdao Biotechnology Co., Ltd. (117°58′58″E, 36°34′41″N). Topsoil (20 cm), collected from five sites (20 m away from each other) inside the greenhouse, was sieved (2-mm sieve) to remove plant debris and rocks. Totally, about 50 kg, soil was prepared and well mixed before cucumber planting. Plant cultivation substrate 1 (PCS1, Organic Matter: 798.6 g kg^−1^, Total N: 17.2 g kg^−1^, NH_4_^+^-N: 113 mg kg^−1^, NO_3_^−^-N: 1,345 mg kg^−1^, Olsen-P: 3,365 mg kg^−1^, NH_4_OAc-K: 18,720 mg kg^−1^, pH: 5.82, and EC: 2.98 mS cm^−1^) was produced by Shandong Xinxile Biotechnology Co., Ltd., with the main component of well-rotted cow dung (85%), and the auxiliary materials of perlite (5%), vermiculite (5%), and coconut coir (5%). Plant cultivation substrate 2 (PCS2, Organic Matter: 792.7 g kg^−1^, Total N: 19.4 g kg^−1^, NH_4_^+^-N:128 mg kg^−1^, NO_3_^−^-N: 1,560 mg kg^−1^, Olsen-P: 2,371 mg kg^−1^, NH_4_OAc-K: 15,628 mg kg^−1^, pH: 6.35, and EC: 2.50 mS cm^−1^) was produced by Hunan Xianghui Agricultural E-commerce Co., Ltd., with straw decomposition as the main component (80%), peat (10%), perlite (5%), and vermiculite (5%) as auxiliary materials. For both artificial cultivation substrates, no additional nutrients were added. For preparing 1× phosphate buffer solution (1× PBS), 8 g NaCl, 0.2 g KCL, 1.44 g Na_2_HPO_4_, and 0.24 g KH_2_PO_4_ were dissolved in 800 ml of distilled water. The pH value was adjusted to 7.4 with HCl, and the volume was fixed to 1 L with water and the solution was autoclaved at 121°C for 20 min. The 1× phosphate buffer solution was prepared for cucumber root sample washing in preparation of cucumber root microbial sequencing samples.

### Planting of Cucumber Seedlings in Different Cultivation Substrates

The experiment was conducted in mid-November 2020 in the same greenhouse at the experimental base of Shandong Biotechnology Co., Ltd. (117°58′58″E; 36°34′41″N). The mean temperature in the greenhouse was kept at 26°C–28°C during the day and 15°C–18°C during the night, with 80%–90% relative humidity. Besides, only natural sunshine illumination with a 10:14-h light/dark cycle was provided, and no plant growth lamp was used during the experiment. The three cultivation substrates were homogenized individually and then filled into pot of 25 cm in diameter and 20 cm in depth, respectively. Each cultivation substrate was filled into 10 pots, and three cucumber seeds were dispersed and sown separately in each pot. After sufficiently watering, the three groups of pots were arranged in cross rows in the middle section of the greenhouse. Once the seeds sprouted and emerged, one cucumber seedling per pot was left to grow. According to previous reports (Yu et al., 2009), the root bacterial abundance of cucumber reached to its peak at its seedling stage, namely about 30 days after sprouting. Thus, the root samples were taken when five true leaves of cucumber were grown (30 days after the seeds sprouted).

### Preparation of Cucumber Root Microbial Sequencing Samples

Six seedlings were randomly selected from each of the three groups of the above cucumber plants. The pot in which the cucumber seedling was planted was cut from the side with scissors, and the seedling with all substrates was taken out. According to the method previously published (Zhang et al., 2021), pieces of cucumber roots were collected and used to access the root microbial community. Specifically, the above-ground part of the cucumber plant was cut off, and the soil or substrate attached to the root was shaken off. Parts of the main, lateral, and adventitious roots were cut with scissors and placed into a sterilized 50-ml centrifuge tube. After collecting root samples from each cucumber seedling, the tools used, such as scissors, were sterilized with 75% ethanol and rinsed thrice with sterilized water to avoid cross-contamination between samples. The centrifuge tubes with root samples were kept in iceboxes and transferred to the laboratory immediately. In total, 18 samples in tubes belonging to three groups were collected: the greenhouse soil group (GHS), plant cultivation substrate 1 group (PCS1), and plant cultivation substrate 2 group (PCS2). Each sample tube received 35 ml of sterilized PBS buffer and shaken at 180 rpm for 20 min. The root samples were then transferred into another tube containing 35 ml of sterilized PBS buffer and shaken as described above. Totally, the root sample was washed thrice. Subsequently, 0.2 g root samples from each tube were ground thoroughly using liquid nitrogen. Total deoxyribonucleic acid (DNA) was extracted using a genomic DNA extraction kit (TIANGEN DP305), and the extracted DNA was examined for quality using a Thermo NanoDrop 2000 micro-UV spectrophotometer.

### High-Throughput Sequencing of Cucumber Root Microbiota

The V5–V7 region of root bacterial 16S rRNA was specifically amplified using primers (Han et al., 2020) including 799F (5′-AACMGGATTAGATACCCKG-3′) and 1193R (5′-ACGTCATCCCCACCTTCC-3′), and the barcode sequence, provided by Shanghai Majorbio Biotechnology Co. Ltd., was added to the 5′ end of the PCR product. The PCR reaction mixture was 50 μl containing 50 ng of total cucumber root DNA template, 1.5 μl of each primer, 5 μl of 2 mmol L^−1^ dNTPs, 2 μl of MgSO_4_, 5 μl of 10× KOD buffer, and 1 μl of KOD Plus, and the remaining volume was made up with ddH_2_O. The PCR conditions were as follows: initial denaturation at 94°C for 2 min; 30 cycles at 94°C for 30 s, 63°C for 30 s, and 68°C for 30 s; and a final extension at 68°C for 5 min. Each cucumber root sample was subjected to three PCR experiments, and the products were subsequently mixed. The PCR products were detected using 2% agarose gel electrophoresis. Then they were purified using the AxyPrep DNA gel recovery kit (AXYGEN). Before the sequence, the DNA in each sample was quantified and diluted to equal concentration. The sequencing of amplicons to generate libraries was accomplished at Shanghai Majorbio Biotechnology Co., Ltd. using the Illumina Miseq PE250 platform.

### High-Throughput Sequencing Data Processing

The Quantitative Insights into Microbial Ecology (QIIME) software package was used for quality assessment of sequencing data and subsequent data analysis (Caporaso et al., 2010). Sequences with lengths below 200 bp, containing ambiguous bases, and primers with more than two bases of mismatches were excluded. The processed sequences were clustered into operational taxonomic units (OTUs) using UPARSE in the USEARCH package according to the 97% sequence similarity criterion (Edgar, 2010). Of the clustered sequences, the sequence with the highest abundance was selected as a representative sequence for this OTU using RDP classifier v.2.2 for taxonomic analysis, and the confidence threshold was set to 70% for taxonomic annotation using the SILVA database (http://www.arb-silva.de; Quast et al., 2013). The OTU composition of each sample at different classification levels was counted to generate an OTU table. One sample from soil (Sample ID: GHS3) determined the fewest sequence reads (7,733 reads). Thus, the sequence data was rarefied to 7,733 reads before further statistical analysis.

Based on the OTU table obtained above, rarefaction curves were generated using “alpha_rarefaction.py” in QIIME to calculate the alpha diversity indices (Sobs, ACE, Chao1, Shannon and Simpson) of the sequenced samples (Schloss et al., 2011). Sobs, Chao1, and Ace can reflect the species richness of the community. The Shannon and Simpson can reflect the species diversity of the community, affected by both species richness and species evenness, that is, the two values also consider the abundance of each species. The Student’s *t* test was used to compare the statistical differences in *α* diversity indices between the two seedling cultivation substrates and soil, respectively. *β* diversity was calculated using non-metric multidimensional scaling (NMDS, based on a Bray–Curtis distance matrix; Clarke, 1993; Hammer et al., 2001) and principal coordinate analysis (PCoA, weighted normalised UniFrac; Chen, 2012). The Student’s *t* test was used to compare the differences in genus abundance between diverse groups of samples.

Functional prediction of cucumber root bacteria was analyzed using the PICRUSt (Langille et al., 2013). The OTU abundance table was first normalized. Subsequently, the COG information and KEGG Ortholog (KO) information corresponding to the OTU were obtained from the Greengene ID, and the corresponding abundance was calculated according to the OTU table. Thus, the information and abundance of the metabolic pathways at three levels was obtained. As mentioned above, the DNA in each sample was quantified and diluted to equal concentration before high-throughput sequencing. Besides, taking the PCR efficiency variation for each sample during sequencing into consideration, obtained sequence data were rarefied to 7,733 reads, which were the reads number of sample from soil (Sample ID: GHS3), before statistical analysis. Thus, each sample contained 7,733 reads. Basing on this, the abundance difference of metabolic pathways at the third level among the three groups was statistically compared with one-way ANOVA. Metabolic pathways at the third level with statistically significant differences in the substrate-cultivated cucumber root bacterial samples compared to soil-cultivated root bacterial samples were screened. The number of the metabolic pathways at the third level in the functional categories of environmental information processing, organismal systems, metabolism and others were counted. The significant upregulation fold of metabolic pathways at third level in plant cultivation substrate 1 and plant cultivation substrate 2 samples with those in greenhouse soil group samples was calculated, and pathways with upregulation fold more than 1.5 were shown.

## Results

### Significant Differences in **α**-Diversity of Root Bacterial Communities Between Substrate-Grown and Soil-Grown Cucumber Seedlings

**Figure 1:**
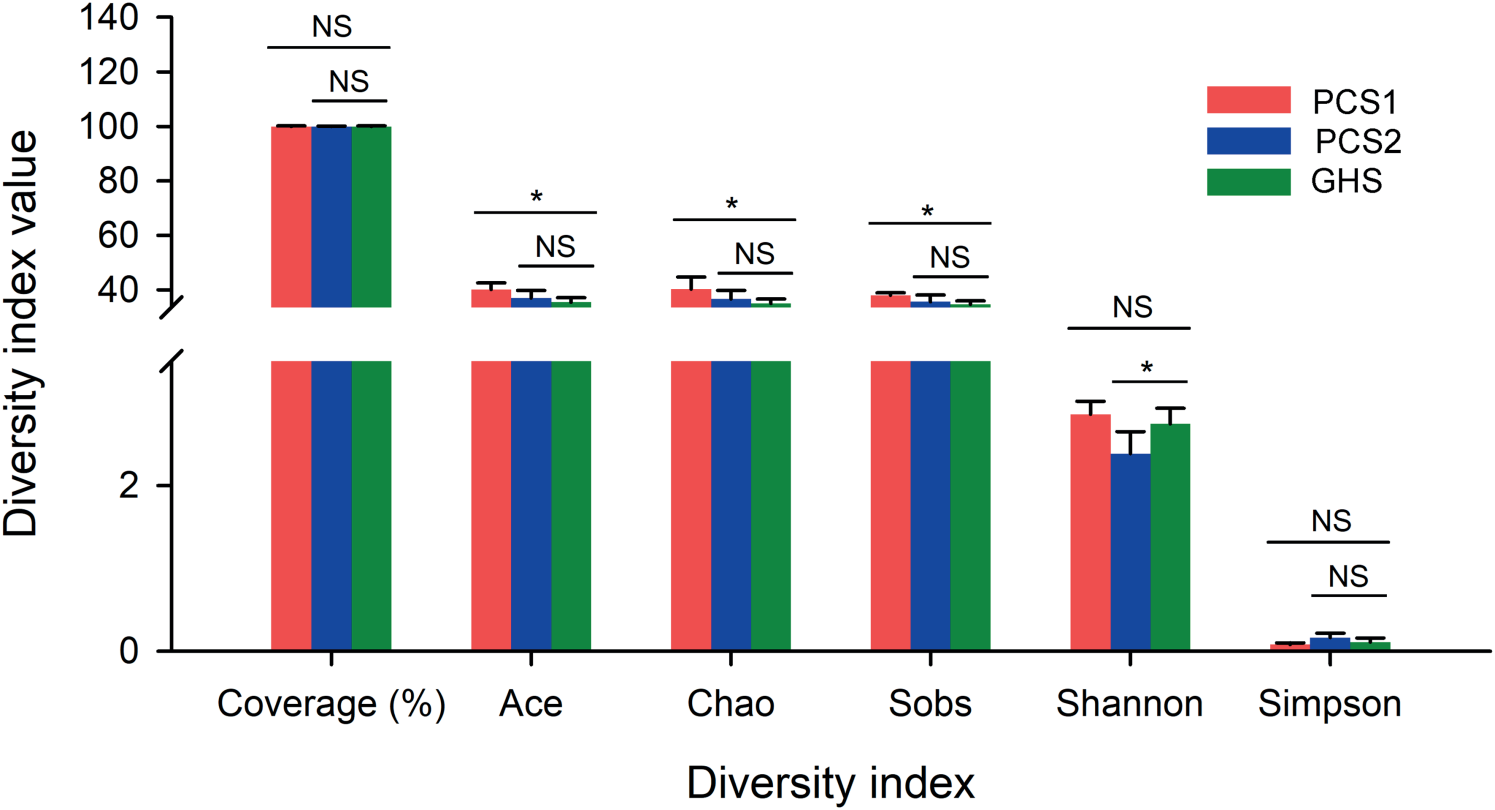
The *α* index of root bacterial microbiota from cucumber grown in different cultivation substrates. “*” in the figure presents significant difference between the connected bars by the line (Student’s *t* test), and “NS” no significant difference. PCS1, cucumber root samples cultivated in plant cultivation substrate 1; PCS2, cucumber root samples cultivated in plant cultivation substrate 2; and GHS, cucumber root samples cultivated in greenhouse soil.

The rarefaction curves of all samples tended to be flat, indicating that the sequencing data in this experiment were sufficient to ensure the reliability of subsequent diversity analysis (Supplementary Figure S1). Besides, the coverage for the three groups of samples almost reached to 100%, and no significant difference were observed among the three groups. Results revealed significant differences between the root samples from the substrate- and the soil-cultivation groups in terms of the richness and evenness of the bacterial communities (Figure 1). Among α-diversity indices, the Ace, Chao, and Sob indices of bacterial communities in the substrate 1 group were significantly higher than those in the soil group, indicating that there were significant differences between the two groups of samples in terms of the number of OTUs, and the species diversity in substrate 1 was higher. Taking the richness and evenness of microbial communities in the samples into consideration, there was no significant difference in microbial diversity between substrate 1 and soil (Shannon and Simpson index). The Ace, Chao, and Sob indices of microbial communities in the samples of the substrate 2 group were not significantly different from those in the soil group, indicating no significant differences in the number of species comprising the microbial communities between the two groups. However, the evenness in the samples of the substrate 2 group was reduced compared with the samples of the soil group (Shannon and Simpson index). Although there were differences in α-diversity among the three cucumber root samples, there was only a slight difference in the values among the three groups in terms of each α-diversity index.

### Root Bacterial Community **β**-Diversity of Substrate-Grown Cucumber Root Samples Differed Significantly From Soil-Grown Samples

**Figure 2:**
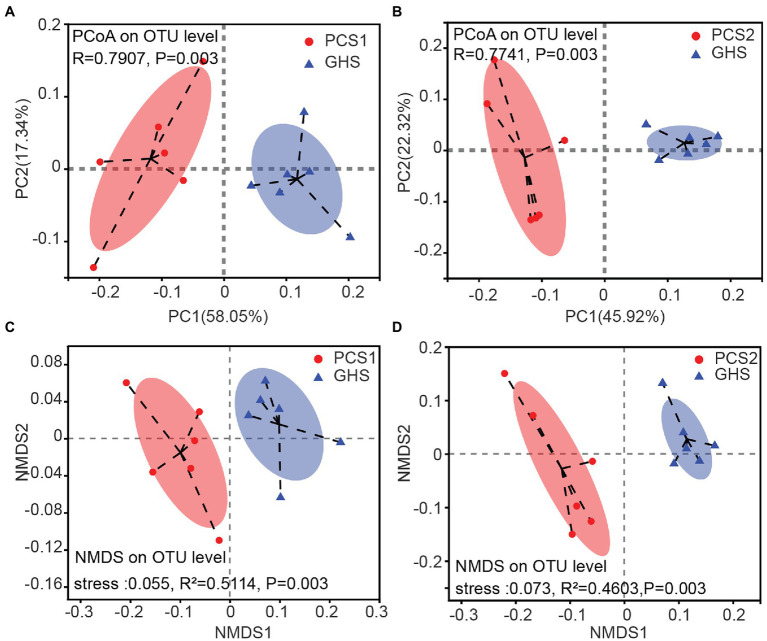
Principal coordinate analysis (PCoA) and non-metric multidimensional scaling (NMDS) plots for root bacterial microbiota of cucumber grown in different cultivation substrates. **(A,B)** PCoA plots, **(C,D)** NMDS plots. PCoA plots are based on the weighted UniFrac metric for microbial communities. Significance values refer to analysis of similarity (Adonis, *p* < 0.05) test for differences in community composition between the two treatments. NMDS diagrams are based on a Bray–Curtis distance matrix for microbial communities that consisted of operational taxonomic units (OTUs; 97% similarity level). Significance values refer to analysis of similarity (ANOSIM, *p* < 0.05) test for differences in community composition between the two treatments. PCS1, cucumber root samples cultivated in plant cultivation substrate 1; PCS2, cucumber root samples cultivated in plant cultivation substrate 2; and GHS, cucumber root samples cultivated in greenhouse soil.

Considering the evolutionary relationships and abundance of species in the microbial community, PCoA revealed that root microbial samples of both the two cultivation substrates clustered separately from the samples of greenhouse soil, each forming a statistically different group (PCS1, Figure 2A, ANOSIM, displacement number = 999, *R* = 0.7907, *p* = 0.003; PCS2, Figure 2B, ANOSIM, displacement number = 999, *R* = 0.7741, *p* = 0.003). The same trend was observed in NMDS analysis, where the two groups of samples from cultivation substrates were clustered separately from the samples of greenhouse soil, each forming a statistically different group (PCS1, Figure 2C, Adonis, displacement number = 999, *R*^2^ = 0.5114, *p* = 0.003; PCS2, Figure 2D, Adonis, displacement number = 999, *R*^2^ = 0.4603, *p* = 0.003). These results indicated that the cucumber root samples from the two cultivation substrates differed significantly in microbial community composition with samples from greenhouse soils.

### Species Composition of Cucumber Root Bacterial Communities From Different Cultivation Substrates Was Almost the Same

**Figure 3:**
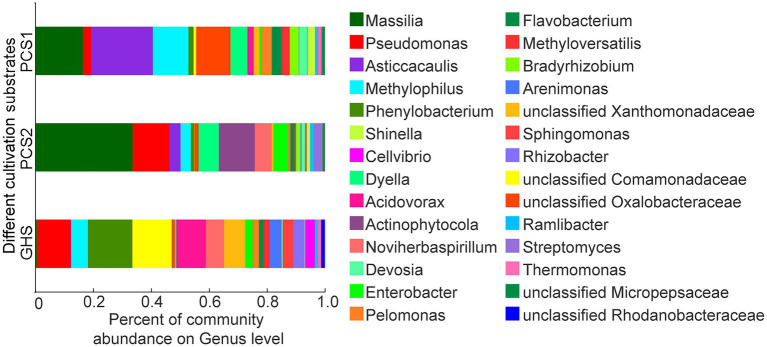
Species composition of root bacterial microbiota on genus level from cucumber grown in different cultivation substrates. PCS1, cucumber root samples cultivated in plant cultivation substrate 1; PCS2, cucumber root samples cultivated in plant cultivation substrate 2; and GHS: cucumber root samples cultivated in greenhouse soil.

Based on sequences annotation, only five bacteria were identified to the species level, including *Flavobacterium anhuiense*, *F. succinicans*, *F. suncheonense*, *Massilia putida*, and *Pseudomonas mosselii*, whereas the others were only annotated to the genus or family level. Therefore, in this study, the species composition in the three groups of samples was analyzed and compared at the genus level. As presented in Figure 3, bacteria belonging to 28 bacterial genera including *Phenylobacterium* (15.35%), unclassified *Comamonadaceae* (13.59%), *Pseudomonas* (11.63%), *Acidovorax* (10.21%), unclassified *Xanthomonadaceae* (7.26%), *Noviherbaspirillum* (6.29%), *Methylophilus* (5.78%), *Arenimonas* (4.23%), *Rhizobacter* (3.94%), *Sphingomonas* (3.63%), *Cellvibrio* (3.40%), *Enterobacter* (2.53%), *Pelomonas* (2.22%), *Methyloversatilis* (1.93%), *Flavobacterium* (1.63%), unclassified *Rhodanobacteraceae* (1.30%), *Thermomonas* (1.18%), unclassified *Oxalobacteraceae* (1.07%), *Ramlibacter* (0.88%), *Dyella* (0.55%), *Massilia* (0.50%), *Devosia* (0.41%), *Asticcacaulis* (0.22%), *Shinella* (0.14%), *Streptomyces* (0.08%), *Actinophytocola* (0.02%), *Bradyrhizobium* (0.01%), and unclassified *Micropepsaceae* (0.01%) were detected in cucumber root samples from soils (GHS). Similarly, bacteria belonging to 27 bacterial genera including *Asticcacaulis* (21.26%), *Massilia* (16.43%), *Methylophilus* (12.31%), unclassified *Oxalobacteraceae* (11.86%), *Dyella* (5.83%), *Pelomonas* (3.62%), *Flavobacterium* (3.47%), *Bradyrhizobium* (3.12%), *Devosia* (2.87%), *Pseudomonas* (2.86%), *Methyloversatilis* (2.73%), *Shinella* (2.46%), *Acidovorax* (1.91%), unclassified *Xanthomonadaceae* (1.90%), *Phenylobacterium* (1.77%), *Thermomonas* (1.35%), unclassified *Micropepsaceae* (1.12%), unclassified *Comamonadaceae* (0.90%), *Ramlibacter* (0.86%), *Enterobacter* (0.63%), *Actinophytocola* (0.22%), *Noviherbaspirillum* (0.18%), *Arenimonas* (0.12%), *Sphingomonas* (0.11%), *Rhizobacter* (0.05%), *Cellvibrio* (0.04%), and *Streptomyces* (0.04%) were detected in cucumber root samples from plant cultivation substrate 1 (PCS1). Besides, bacteria belonging to 26 bacterial genera including *Massilia* (33.61%), *Pseudomonas* (12.71%), *Actinophytocola* (12.14%), *Dyella* (7.06%), *Noviherbaspirillum* (5.83%), *Enterobacter* (5.02%), *Asticcacaulis* (3.80%), *Methylophilus* (3.54%), *Streptomyces* (2.73%), *Bradyrhizobium* (1.56%), *Flavobacterium* (1.43%), unclassified *Oxalobacteraceae* (1.43%), *Devosia* (1.22%), *Ramlibacter* (1.22%), *Phenylobacterium* (1.18%), *Shinella* (1.15%), unclassified *Micropepsaceae* (0.88%), *Pelomonas* (0.85%), unclassified *Xanthomonadaceae* (0.60%), *Sphingomonas* (0.59%), *Methyloversatilis* (0.48%), *Arenimonas* (0.34%), *Thermomonas* (0.28%), *Acidovorax* (0.26%), unclassified *Comamonadaceae* (0.06%), and *Rhizobacter* (0.03%) were detected in cucumber root samples from plant cultivation substrate 2 (PCS2). The root bacterial communities of cucumber in the three cultivation substrates predominantly included certain genera, including *Acidovorax*, *Actinophytocola*, *Arenimonas*, *Asticcacaulis*, *Bradyrhizobium*, *Cellvibrio*, *Devosia*, *Dyella*, *Enterobacter*, *Flavobacterium*, *Massilia*, *Methylophilus*, *Methyloversatilis*, *Noviherbaspirillum*, *Pelomonas*, *Phenylobacterium*, *Pseudomonas*, *Ramlibacter*, *Rhizobacter*, *Shinella*, *Sphingomonas*, *Streptomyces*, *Thermomonas*, and families including *Comamonadaceae*, *Oxalobacteraceae*, *Rhodanobacteraceae*, *Xanthomonadaceae*, and *Micropepsaceae*. The abundance of each genus in the root bacteria may vary slightly between the substrate and soil group samples.

**Figure 4:**
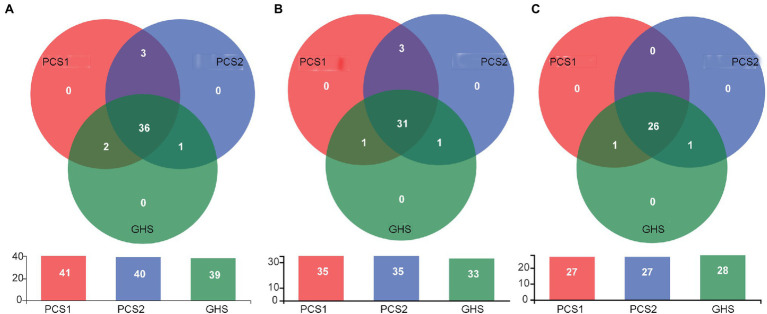
Venn diagram for root bacterial microbiota composition of cucumber grown in different cultivation substrates. **(A)** OTU level, **(B)** Species level, and **(C)** Genus level. PCS1, cucumber root samples cultivated in plant cultivation substrate 1; PCS2, cucumber root samples cultivated in plant cultivation substrate 2; and GHS, cucumber root samples cultivated in greenhouse soil.

Subsequently, the species composition of bacterial microbiota from cucumber roots cultivated in different substrates was compared. At the OTU level (Figure 4A), cucumber root samples from the substrate 1, substrate 2, and soil groups contained 41, 40, and 39 OTUs, respectively. Of these groups, a total of 38 OTUs were common in the substrate 1 and soil groups, with three OTUs specific to the substrate 1 group and one OTU specific to the soil group. Additionally, a total of 37 OTUs were common in the substrate 2 and soil groups, with three OTUs specific to the substrate 2 group and two OTUs specific to the soil group. At the species level (Figure 4B), cucumber root samples from the substrate 1, substrate 2, and soil groups contained 35, 35, and 33 species, respectively (some of which were not accurately identified to the species level). Of these groups, substrate 1 and soil shared 32 species, with three species specific to substrate 1 (*Asticcacaulis*_sp. 1, *Asticcacaulis* sp. 2, and *F. suncheonense*) and one endemic to soil (unclassified *Rhodanobacteraceae*). There were 32 species shared by both the substrate 2 and soil groups, with three species endemic to substrate 2 (*Asticcacaulis* sp. 1, *Asticcacaulis* sp. 2, and *F. suncheonense*) and one species endemic to soil (*Cellvibrio* sp.). The 31 species that the three groups of samples had in common were as follows: *Massilia putida*, *Pseudomonas* sp., *Devosia* sp., *Methylophilus* sp., *F. succinicans*, *Thermomonas* sp., uncultured *Micropepsaceae*, *Phenylobacterium* sp. 1, *Asticcacaulis* sp., *Methyloversatilis* sp., *Rhizobacter* sp., *Phenylobacterium* sp. 2, unclassified *Oxalobacteraceae*, *F. anhuiense*, *Streptomyces* sp., *Dyella* sp., *Enterobacter* sp., unclassified *Xanthomonadaceae*, *Bradyrhizobium* sp., *P. mosselii*, *Sphingomonas* sp., *Noviherbaspirillum* sp., *Telluria* sp., *Acidovorax* sp., *Pelomonas* sp., *Shinella* sp., unclassified *Comamonadaceae*, *Arenimonas* sp., *Ramlibacter* sp., *Arenimonas* sp., and *Actinophytocola* sp. At the genus level (Figure 4C), cucumber root samples from the substrate 1, substrate 2, and soil groups contained 27, 27, and 28 genera, respectively. A total of 27 genera were common between the substrate 1 and soil groups, with no genus endemic to substrate 1 and one genus endemic to soil (unclassified *Rhodanobacteraceae*) In addition, 27 genera were common to the substrate 2 and soil groups, with no endemic genus to substrate 1 and one soil-specific genus (*Cellvibrio*). The 26 genera common among all three sets of samples included *Devosia*, *Dyella*, *Ramlibacter*, *Enterobacter*, *Massilia*, unclassified *Micropepsaceae*, unclassified *Comamonadaceae*, *Pelomonas*, *Asticcacaulis*, unclassified *Xanthomonadaceae*, *Flavobacterium*, *Methyloversatilis*, *Sphingomonas*, *Phenylobacterium*, *Arenimonas*, *Thermomonas*, unclassified *Oxalobacteraceae*, *Methylophilus*, *Actinophytocola*, *Rhizobacter*, *Acidovorax*, *Bradyrhizobium*, *Streptomyces*, *Noviherbaspirillum*, *Shinella*, and *Pseudomonas*.

### Genus Abundance in Root Bacterial Communities of Cucumber From Different Cultivation Substrates Were Significantly Different

**Figure 5:**
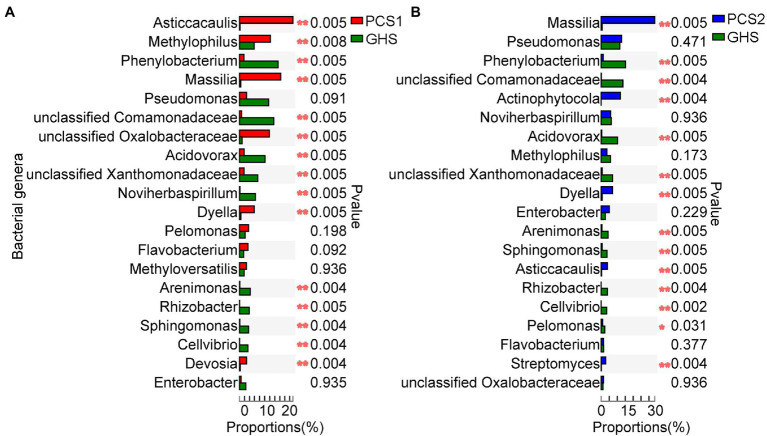
Comparison of genus abundance in root bacterial microbiota from cucumber root samples grown in different cultivation substrates. **(A)** Comparison of genus abundance (Top 20) in root bacterial microbiota from cucumber root samples grown in plant cultivation substrate 1 (PCS1) and greenhouse soil (GHS). **(B)** Comparison of genus abundance (Top 20) in root bacterial microbiota from cucumber root samples grown in plant cultivation substrate 2 (PCS2) and greenhouse soil (GHS). “*” at the right of each group of bars in **(A,B)** present significant difference (^*^p < 0.05; ^**^p < 0.01; and Student’s *t* test).

Among the top 20 genera in terms of abundance in the bacterial community of substrate 1 root samples, *Asticcacaulis*, *Methylophilus*, *Massilia*, *Dyella*, *Devosia*, and a genus of the family *Oxalobacteraceae* were significantly more abundant whereas the genera *Phenylobacterium*, *Acidovorax*, *Noviherbaspirillum*, *Arenimonas*, *Rhizobacter*, *Sphingomonas*, and *Cellvibrio* and the families *Comamonadaceae* and *Xanthomonadaceae* were significantly less abundant in the substrate 1 samples than in the soil samples (Figure 5A). Among the top 20 genera in terms of abundance, the genera *Massilia*, *Actinophytocola*, *Dyella*, *Asticcacaulis*, and *Streptomyces* were significantly more abundant whereas the genera *Phenylobacterium*, *Acidovorax*, *Arenimonas*, *Sphingomonas*, *Rhizobacter*, *Cellvibrio*, and *Pelomonas* and two genera of the *Comamonadaceae* and *Xanthomonadaceae* families were significantly less abundant in the substrate 2 samples than in the soil samples (Figure 5B).

### Abundance of Metabolic Pathways in the Root Bacterial Communities of Cucumber in Different Cultivation Substrates Was Significantly Different

**Figure 6:**
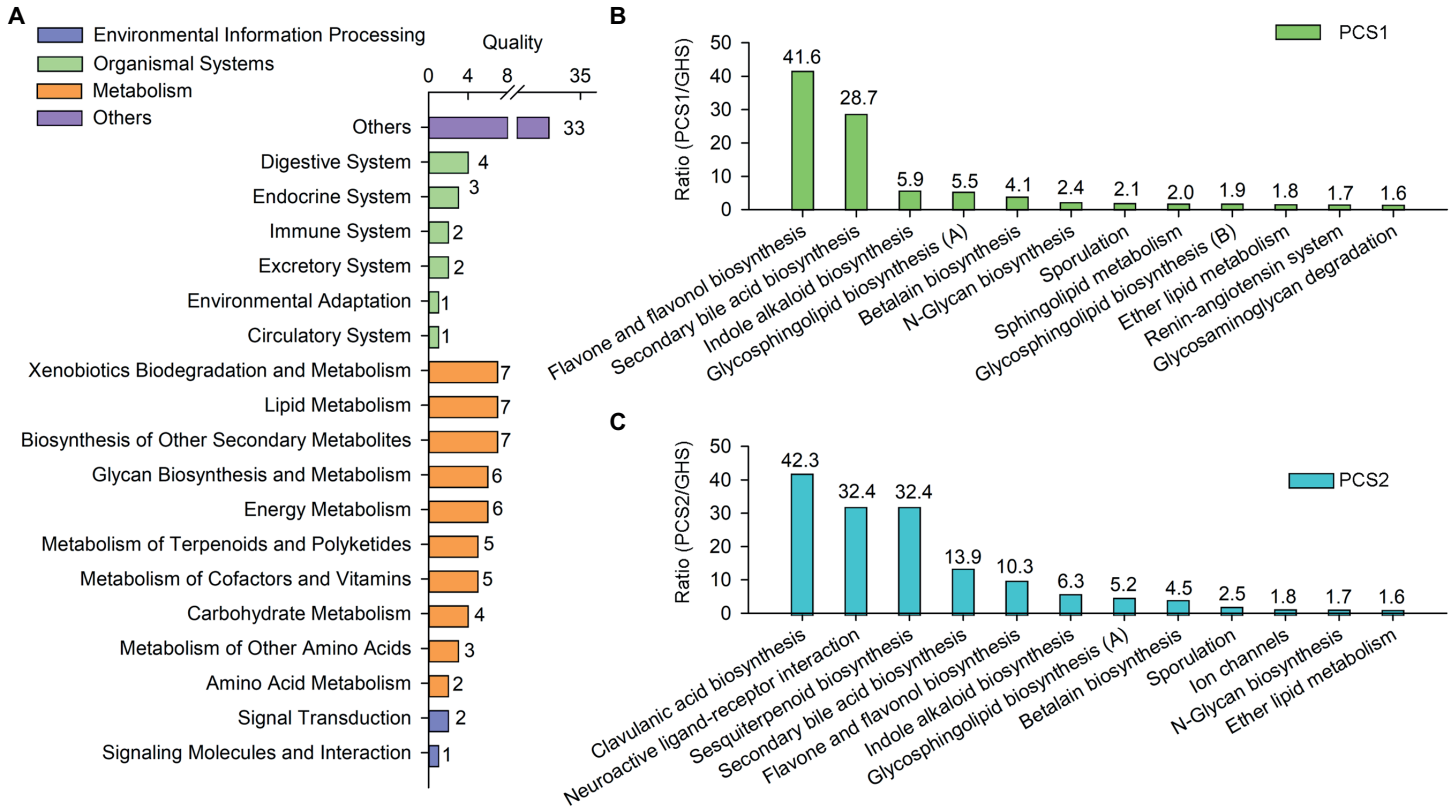
Metabolic function of root bacterial microbiota from cucumber grown in different cultivation substrates. **(A)** Quality of metabolic pathways in root bacterial microbiota, which were significantly different among the two plant cultivation substrates and greenhouse soils. **(B)** Upregulated metabolic pathways (>1.5) in root bacterial microbiota from substrate 1-cultivated (PCS1) cucumber roots compared to that from greenhouse soil (GHS). **(C)** Upregulated metabolic pathways (>1.5) in root bacterial microbiota from substrate 2-cultivated (PCS2) cucumber roots compared to that from soil (GHS).

The abundance of 101 metabolic pathways in the root bacterial community of cucumber seedlings cultivated in different substrates were significantly different (Figure 6A), mainly including environmental information processing (three pathways), organismal systems (13 pathways), metabolism (52 pathways), and other functions (33 pathways). Among the metabolism pathways, the synthetic pathways of certain metabolites were primarily involved, such as the metabolism of amino acids, carbohydrates, cofactors and vitamins, terpenoids and polyketides, secondary metabolites, lipids, and other compounds etc. (Figure 6A). Compared to root bacterial community of cucumber roots cultivated in soils, 12 metabolic pathways in the bacterial community of the substrate 1-cultivated cucumber roots exceeded the soil group in abundance, with a significant upregulation of more than 1.5-fold (Figure 6B), including the synthetic pathways of flavonoids and flavonols, bile acids, indole alkaloids, lactose, and neolactose (glycosphingolipid biosynthesis [A]), which increased in abundance by 41.6-, 28.7-, 5.9-, and 5.5-fold, respectively. Besides, the abundance of 12 metabolic pathways in the bacterial community of substrate group 2 significantly exceeded that of the soil group. The pathways were significantly upregulated by more than 1.5-fold (Figure 6C), with the abundance of clavulanic acid, receptor interaction, sesquiterpenoid, bile acid, flavonoid and flavonol, indole alkaloid, lactose, and neolactose (glycosphingolipid biosynthesis [A]) synthetic pathways increased by 42.3-, 32.4-, 32.4-, 13.9-, 10.3-, 6.3-, and 5.2-fold, respectively.

## Discussion

In recent years, the bacterial microbiota of crops have been manipulated to improve crop fitness (Kwak et al., 2018), which in turn promoted the investigation of crop associated microbiota. In this study, the bacterial diversity of cucumber root cultivated in different substrates was examined. In total, bacteria belonging to 28 genera, including *Phenylobacterium*, unclassified *Comamonadaceae*, *Pseudomonas, Acidovorax*, unclassified *Xanthomonadaceae*, *Noviherbaspirillum*, *Methylophilus*, *Arenimonas*, *Rhizobacter*, *Sphingomonas*, *Cellvibrio*, *Enterobacter*, *Pelomonas*, *Methyloversatilis*, *Flavobacterium*, unclassified *Rhodanobacteraceae*, *Thermomonas*, unclassified *Oxalobacteraceae*, *Ramlibacter*, *Dyella*, *Massilia*, *Devosia*, *Asticcacaulis*, *Shinella*, *Streptomyces*, *Actinophytocola*, *Bradyrhizobium*, and unclassified *Micropepsaceae* were detected. The abundance of *Massilia*, *Methylophilus*, *Phenylobacterium*, *Pseudomonas*, *Dyella*, *Acidovorax*, and *Actinophytocola* was high in cucumber root bacterial community samples (Figure 3), which indicates that they might be dominant bacterial genera of cucumber root bacteria. Some of the above bacterial clades have been reported in previous studies. For example, bacteria of the genera *Pseudomonas* and *Streptomyces* are present in the rhizosphere of some greenhouse cucumbers (Gao, 2019). In addition, bacteria from genus *Devosia*, *Dyella*, *Massilia*, *Asticcacaulis*, *Flavobacterium*, *Methyloversatilis*, *Sphingomonas*, *Rhizobacter*, *Acidovorax*, *Bradyrhizobium*, *Streptomyces*, *Noviherbaspirillum*, *Shinella*, and *Pseudomonas* were observed in the rhizosphere of cucumber seedlings grown in artificial substrate (Dong et al., 2018). Generally, plant roots produce specific secretions that allow some specific species of fungi or bacteria to multiply in the rhizosphere and consequently form the core microflora of rhizosphere microorganisms (Doornbos et al., 2011; Wen et al., 2020). Except being affected by the plant host, the root microbiome is also affected by the cultivation environment owing to differences in the physicochemical properties of cultivation substrates (Shao et al., 2021). As it was shown in Figure 4A, among the totally detected 39–41 OTUs in samples from both greenhouse soils and plant cultivation substrates, 36 OTUs were shared by root samples from three groups. Similar results were also obtained on bacterial species (Figure 4B) and genus (Figure 4C) level. Thus, bacterial species detected in this study are likely to belong to the core group of cucumber root bacteria, which may not change with cucumber cultivation conditions as had been reported in *Arabidopsis thaliana* (Lundberg et al., 2012). However, this work only focused on one variety of cucumber and one growth stage, which may not reveal the full view of cucumber root core microbiota, further investigation on the core root bacterial microbiota of cucumber needs to be conducted by taking factors such as the cultivation geography and cucumber varieties into consideration.

Furthermore, this study observed that the root bacterial communities of cucumber cultivated in the three cultivation substrates shared majority of the detected bacterial species (Figures 3, 4), although both the corresponding α- and β-diversity were significantly different (Figures 1, 2). The number of species composition at the OTU, species and genus levels did not differ dramatically among the three cultivation substrates, with only approximately 2–3 species specific to the three cultivation substrates (Figure 4). This may be owing to two reasons. On one hand, in the process of preparing sequencing samples of cucumber roots, we cleaned the cucumber roots based on a previous protocol (Zhang et al., 2021) to wash off the non-root bacteria adhered to the roots to prevent adulteration of the cucumber root bacterial community with the bacteria from cultivation substrates. Consequently, a stable cucumber root microbiota including both some rhizospheric and endophytic bacteria was obtained. On the other hand, the cucumber root secretion species are relatively stable (Xu, 2006). Only specific species of bacterial phyla in the cultivation substrates and species carried by cucumber seeds can be enriched and colonize in cucumber roots under the influence of secretions, although different species of bacteria may exist in the cultivated substrates. Therefore, as proposed by previous studies, the root bacterial species of cucumber in different cultivation substrates were relatively stable (Muller et al., 2016).

Although the cucumber root bacterial species composition among the three cultivation substrates was almost the same, the abundance of dominant bacterial genera differed significantly between the two substrates and soil (Figure 4). For example, genera such as *Phenylobacterium* and *Acidovorax* were significantly more abundant in soil-cultivated cucumber than in cucumber cultivated in both cultivation substrates, whereas the bacteria of genera such as *Massilia* and *Dyella* were more abundant in both substrates. Several factors may lead to this phenomenon. First, there are major differences in nutrient composition between artificial substrates and soil, and such differences can lead to changes in the types of root secretions of cultivated crops (Chen et al., 2020b), which can directly lead to changes in the root bacterial flora (Doornbos et al., 2011). Second, the differences in the physical and chemical properties of substrates and soils may lead to variation in the abundance of certain bacterial genera. For example, the main component of plant cultivation substrate 2 was straw; whose microbial metabolites such as organic acids decrease the overall pH of the substrate (Cui et al., 2020). Consequently, the abundance of certain specific genera of bacteria such as *Acidovorax* who grow well on acidic media would increase (Willems et al., 1990). Besides, there are large differences between the physical structure of artificial cultivation substrates and soil. Generally, the soil is dense and with a relatively tight physical structure, while the artificial cultivation substrate is relatively loose in physical structure and with large voids within it. This difference is likely to lead to disparities in oxygen levels around the roots during cultivation, which in turn leads to differences in the abundance of some bacteria. For example, the aerobic genera *Massilia* (Yang et al., 2019) and *Asticcacaulis* (Vasilyeva et al., 2006) were significantly more abundant in the artificial cultivation substrate than in the soil-cultivated samples, whereas the facultative anaerobic bacteria *Phenylobacterium*, *Arenimonas*, and *Sphingomonas* (Anjum et al., 2018) were significantly more abundant in the soil group samples than in the substrate-cultivated cucumber roots. Third, differences in nutrients in the artificial cultivation substrate and soil may have contributed to the significant differences in the abundance of the cucumber root bacterial genera. The main component of the selected cultivation substrates in this study is organic matter, which may affect the microbial community especially the abundance of specific genera according to previous reports (Tian and Gao, 2014).

Corresponding to the abundance of bacterial genera, certain metabolic pathways including flavonoids and flavonols, bile acids, indole alkaloids, lactose and neolactose, clavulanic acid, and sesquiterpenoid were significantly upregulated in the root bacterial communities of cucumber grown in both artificial substrates compared with those grown in soils (Figure 6). According to previous studies, flavonoids are important in promoting plant growth, especially root growth (Khalid et al., 2019), and indoles are essential in the synthesis of plant growth hormones (Baumann et al., 2017). In addition, sesquiterpenoids are effective in promoting plant growth and resistance to various adversities (Chen et al., 2020a). Accordingly, the shift of metabolic pathways mentioned above may contribute to promoted growth of cucumber in artificial cultivation substrates than in soils, which was observed during the experiment (data not provided). Furthermore, the abundance of certain genera such as *Massilia* (Yang et al., 2019) and *Asticcacaulis* (Okazaki et al., 2021) with growth-promoting, phosphorus and potassium solubilizing functions were significantly increased in the root samples of cucumber cultivated in artificial substrates, which is consistent with the functional predictions. In conclusion, metabolic pathways associated with the synthesis of the above-mentioned compounds were upregulated in the root microbial community of cucumber cultivated with artificial substrates, and this may promote the growth of cultivated cucumber.

## Conclusion

High-throughput sequencing revealed that cucumber root bacteria cultivated with two substrates and soil had significant differences in α-diversity and β-diversity, which were mainly owing to the differences in species abundance instead of species composition. In addition, bacterial species with growth-promoting effects was more enriched in cucumber root cultivated with the artificial substrate. Therefore, this study provides a theoretical basis for exploring beneficial microorganisms from the cucumber rhizosphere microbiome and constructing artificial bacterial communities to guide agricultural production in the future.

## Data Availability Statement

The datasets presented in this study can be found in online repositories. The names of the repository/repositories and accession number(s) can be found at: NCBI BioProject—PRJNA814873.

## Author Contributions

FZ was responsible for the conceptualization, methodology, software, data curation, and writing—original draft preparation. XW was responsible for visualization and investigation. YG was responsible for the investigation. SF was responsible for writing—reviewing and editing. HZ was responsible for the investigation. XZ was responsible for supervision, software, validation, and writing—reviewing and editing. All authors contributed to the article and approved the submitted version.

## Funding

This work was funded by Major Scientific and Technological Innovation Projects in Shandong Province (2019JZZY020610 and 2021TZXD002) and the New Twenty Policies for University in Jinan Project (2021GXRC040).

## Conflict of Interest

The authors declare that the research was conducted in the absence of any commercial or financial relationships that could be construed as a potential conflict of interest.

## Publisher’s Note

All claims expressed in this article are solely those of the authors and do not necessarily represent those of their affiliated organizations, or those of the publisher, the editors and the reviewers. Any product that may be evaluated in this article, or claim that may be made by its manufacturer, is not guaranteed or endorsed by the publisher.
