# Supplementary figures and images for: Diversity Shifts in the Root Microbiome of Cucumber Under Different Plant Cultivation Substrates
Source: Front Microbiol. 2022 May 19;13:878409. doi: 10.3389/fmicb.2022.878409 (PMC9159939; doi:10.3389/fmicb.2022.878409)

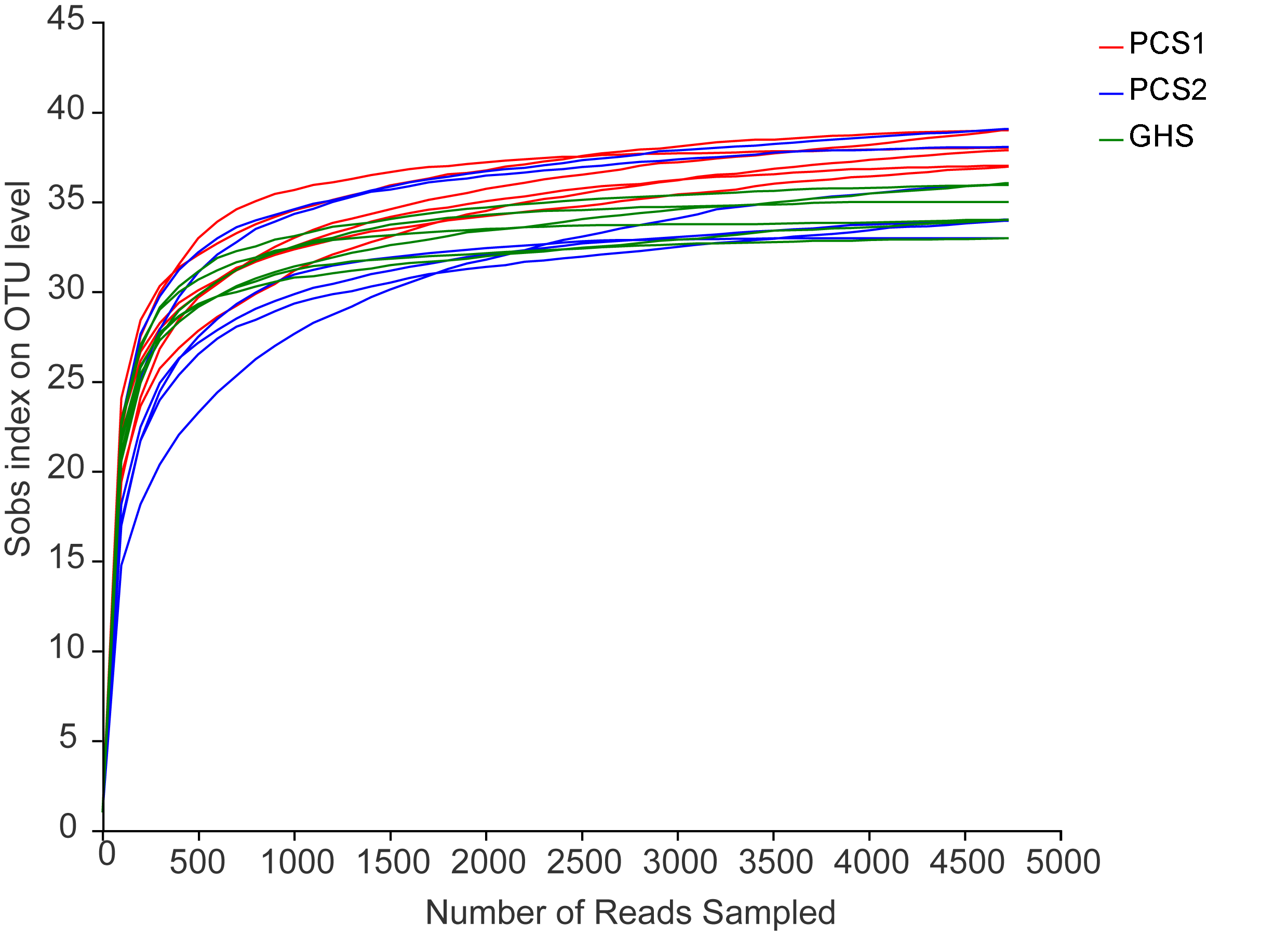

Supplement: Supplementary file 1 [file Image_1.TIF]
